# Effects of Biochar With Inorganic and Organic Fertilizers on Agronomic Traits and Nutrient Absorption of Soybean and Fertility and Microbes in Purple Soil

**DOI:** 10.3389/fpls.2022.871021

**Published:** 2022-03-25

**Authors:** Ming Liu, Cholidah Linna, Shumin Ma, Qun Ma, Jinge Guo, Fenfen Wang, Longchang Wang

**Affiliations:** College of Agronomy and Biotechnology, Southwest University, Chongqing, China

**Keywords:** biochar, inorganic fertilizer, organic fertilizer, soybean, soil microbial community

## Abstract

Biochar is a kind of organic matter that can be added into the soil as a soil amendment to improve its quality. What are the effects of using biochar on purple soil and soybeans? Can the use of biochar reduce the use of fertilizers? This is our concern. Therefore, we carried out this study. The objectives of our study were to evaluate the effects of biochar, inorganic and organic fertilizer application on plant growth, chlorophyll content, photosynthetic gas exchange, and yield of soybean as well as fertility and microbial community in purple soil, and to appraise the possible reduction rate of inorganic fertilizer under the biochar application. A pot experiment was conducted with three levels of biochar, two levels of inorganic fertilizer, and two levels of organic fertilizer in a randomized complete block. The results indicated that the low rate of biochar together with half rate of inorganic fertilizer and organic fertilizer increased the plant growth of soybean. Meanwhile, the chlorophyll content, root growth, and yield of soybean were increased by 16.61, 197.73, and 96.7%, respectively, with biochar compared with no biochar. The high rate of biochar with half rate of inorganic fertilizer and organic fertilizer can promote the exchange of photosynthetic gas in soybean, and the photosynthetic rate increased by 45.25% compared with the blank control. At the full pod stage, the nitrogen content, phosphorus content, and potassium content of the whole plant under the high rate of biochar were 28.35, 13.65, and 28.78%, respectively, higher than that of the blank control. The application of biochar increased nitrogen, phosphorus, and potassium uptake of soybean. The high rate of biochar with half rate of inorganic fertilizer and organic fertilizer can improve soil nutrient content and soil microbial community. Compared with no biochar treatments, total organic carbon (TOC) increased by 740.28%, and cation exchange capacity (CEC) increased by 54.17%. Phospholipid fatty acid (PLFA) increased by 65.22%, and all kinds of soil microorganisms increased to varying degrees. In conclusion, the application of biochar can reduce the use of organic and inorganic fertilizers, improve the agronomic traits and yield of soybean, and play a positive role in soil nutrients and soil microorganisms.

## Introduction

At present, the global population is close to 7.5 billion, and it is expected to double to 9.5 billion by 2050 (Lal and Stewart, 2015). Population growth forced to increase the agricultural production to meet food needs (FAO et al., 2015; Jones and Ejeta, 2016). In general, agricultural production is a factor supporting food security, especially food supply (Berry et al., 2015).

Biochar is a stable carbon material produced by pyrolysis of raw materials (O’Laughlin and McElligott, 2009; Woolf et al., 2010; DeLuca et al., 2015). Due to its potential to mitigate climate change, it is conducive to food security and undertakes the management of organic waste, and has significantly been developed worldwide (Nsamba et al., 2015). Over the past decade, the application of biochar has shown its advantages in soil properties, crop growth, and environmental protection (Xu et al., 2012; Carter et al., 2013; Stavi and Lal, 2013; Wilson, 2014; Oliveira et al., 2017; Gayathri et al., 2021; Nguyen et al., 2021). However, soil conditions and fertilizer management affect the utilization of biochar in soil (Jha et al., 2010). Yamato et al. (2006) showed that the application of biochar and inorganic fertilizer could significantly increase the yields of maize, peanut, and cowpea in the barren soil environment. Similarly, Coumaravel et al. (2015) also pointed out that the application of biochar on maize must be combined with the recommended dose of nitrogen, phosphorus, and potassium fertilizers, and must be combined with plant nutrition systems to improve maize productivity. Research by Khan et al. (2021) has shown that the application of biochar can promote growth by increasing chlorophyll content in wheat, thereby increasing yield. Wilson (2014) further explained that from the past to the present, there are several processes in which biochar acts in the soil, with numerous and diverse layers. Zhang et al. (2012) found that the yield of maize was increased by 8.8 and 12.1% under 20 and 40 t ha^–1^ biochar, respectively, combined with nitrogen fertilizer. A recent study by Wang et al. (2016) showed that 80 t ha^–1^ biochar improved the plant growth of soybean. Meanwhile, biochar can also improve the structure of soil farmland microorganisms. Jin et al. (2019) showed that the microbial biomass carbon and nitrogen in farmland soil peaked in the third year of biochar application. Xu et al. (2014) showed that the addition of biochar can cause changes in soil microbial communities. Chen et al. (2016) found in their study on rice that under high-dose biochar application, the growth of some bacterial populations was stimulated, which was significantly higher than that of the control group. Yan et al. (2019) showed that biochar has an impact on the purple soil carbon environment and soil microbial abundance, which may in turn improve soil carbon pools.

Soybean (*Glycine max* L.) is an important crop with multiple uses, such as staple food, bioenergy, animal milk feed, and raw materials for various household industries (Wilson, 2012). At present, due to the lack of advanced technology and management methods, the lack of improved varieties, and the impact of natural resource degradation and climate change, soybean yield is in a bottleneck state (Meador and Xinping, 2014; Pagano and Miransari, 2016). Mete et al. (2015) pointed out that the application of biochar and NPK fertilizers could increase the total biomass and seed yield of soybean in alkaline soils. Maulana et al. (2021) considered that the application of biochar might be one of the ways to improve soybean growth. Studies of biochar on soil properties and plant growth have widely been published. In China, there are about 20 million hectares of purple soil, which is mainly distributed in southwest regions (He et al., 2009). The application of biochar may affect the photosynthetic process of plants, which may be an important mechanism affecting plant production.

The purpose of this study was to evaluate the effects of biochar, inorganic and organic fertilizers on plant growth, photosynthesis, and soybean yield in purple soil, to explore the possible reduction rate of inorganic fertilizer under biochar application, and to evaluate the effects of biochar combined with inorganic fertilizer and organic fertilizer on soil quality and soil microbial community. It will provide physiological insights into the agronomic benefit mechanism of biochar utilization.

## Materials and Methods

### Experimental Area

The research of biochar, inorganic fertilizer, and organic fertilizer application on soybean was conducted using pot experiment in the greenhouse of the College of Agronomy and Biotechnology, Southwest University, China, from April to July 2017. The research area was located at 220 m in altitude, 29°49′32″N in latitude, and 106°26′02″E in longitude. Measurement of soil, biochar, and plant parameters was carried out in the Key Laboratory of Crop Quality Improvement of Chongqing Municipality, College of Agronomy and Biotechnology, Southwest University, China. The soil of the experimental site is dryland purple soil with a gentle slope and relatively uniform soil fertility. The main physical and chemical properties of the soil include the soil bulk density of 1.21 g⋅cm^–3^, the pH value of 6.47, the organic matter content of 28.00 g⋅kg^–1^, the total nitrogen content of 1.68 g⋅kg^–1^, the total phosphorus content of 1.46 g⋅kg^–1^, the total potassium content of 34.54 g⋅kg^–1^, the available phosphorus of 18.13 mg⋅kg^–1^, the available potassium of 270.23 mg⋅kg^–1^, and the alkaline hydrolyzable nitrogen of 35.23 mg⋅kg^–1^.

### Experimental Design

A pot experiment was conducted in randomized complete block design (RCBD) with 3 × 2 × 2 treatments, with three replications. The first treatment was the rate of biochar (B), namely, B0, B1, and B2. The second treatment was fertilizer (F), namely, F1 and F2. The third treatment was organic fertilizer (M), namely, M0 and M1. Table 1 shows the twelve treatment combinations.

**TABLE 1:** Treatment combinations of biochar, inorganic fertilizer, and organic fertilizer.

Treatment	Half dosage of inorganic fertilizer (30 N, 87.5 P_2_O_5_, 60 K_2_O) (kg/ha) (F1)		Half dosage of inorganic fertilizer (60N, 175 P_2_O_5_, 120 K_2_O) (kg/ha)(F2)	
		
	Organic fertilizer 0 t/ha(M0)	Organic fertilizer 4.5 t/ha(M1)	Organic fertilizer 0 t/ha(M0)	Organic fertilizer 4.5 t/ha(M1)
Biochar 0 t/ha (B0)	B0F1M0	B0F1M1	B0F2M0	B0F2M1
Biochar 35t/ha (B1)	B1F1M0	B1F1M1	B1F2M0	B1F2M1
Biochar 50 t/ha (B2)	B2F1M0	B2F1M1	B2F2M0	B2F2M1

The total weight of soil, biochar, and organic fertilizer in each pot was 5,000 *g*. They were mixed with inorganic fertilizer as treatment before sowing. The seeds of soybean (variety: Zhechun No.3) were provided by the Soybean Research Institute, College of Agronomy and Biotechnology, Southwest University, China. The seeds of soybean were inoculated with rhizobium before planting. The seeds were sown on April 15, 2017. Ten seeds were sown in each pot, and thinning was performed at the first true leaf stage to maintain two plants per pot. Soybeans were watered once every 5 days according to the daily temperature. The amount of water added in each pot was the same, i.e., 200–600 ml. The temperature inside the glasshouse ranged from 25.5 to 45°C, and relative humidity (RH) was 30–80% during the entire growth.

Biochar (carbonized corncob) was commercially obtained from Nanjing Qinfeng Straw Technology Co., Ltd., China. Based on the information from the manufacturer, the corncob was prepared at a pyrolysis temperature of 400°C. Inorganic fertilizers used were urea, superphosphate, and potassium chloride as sources of mineral nitrogen, phosphate, and potassium, respectively. Organic fertilizer was a biological organic fertilizer that was commercially obtained and contained more than 50% organic matter. The tested purple soil was collected from the Experiment Farm of Southwest University, China. The soil was air-dried and sieved to pass through 2 mm. The plastic pots used in the experiment were 23 cm in diameter with a total soil volume of 5,000 *g*. The basic physical and chemical properties of biochar include the pH of 9.90 ± 0.04, the electrical conductivity (EC) of 0.94 ± 0.01 mS⋅cm^–1^, the total nitrogen of 5.76 ± 0.23 g⋅kg^–1^, the total phosphorus of 3.75 g⋅kg^–1^, the total potassium of 21.15 ± 0.17 g⋅kg^–1^, the cation exchange capacity (CEC) of 34.49 ± 5.16 cmol⋅kg^–1^, and the organic carbon of 439.01 ± 40.06 g⋅kg^–1^.

### Growth, Yield, and Yield Components

The data of soybean plants were collected at four stages of growth, namely, vegetative stage (V6), beginning pod stage (R3), full pod stage (R6), and full maturity stage (R8). Leaf area was measured with the gravimetric method. Plant height was measured with a ruler. Stem diameter was measured with a caliper. Fresh and dry weights of plants were measured gravimetrically with an electronic scale.

Soybean plants were harvested at the end of July 2017. The plants were removed from the pot and divided into stems and pods, while roots were washed thoroughly. Dry matter of plant samples was obtained after being dried in an oven at 65°C for 48 h. The ratio of grain yield to biological yield was calculated as harvest index (HI). Leaf area index (LAI) was calculated by:

LAI = La/A

where La is leaf area (cm^2^) and A is ground area (cm^2^).

### Chlorophyll Assessments

Chlorophyll contents, including chlorophyll a (Chl a), chlorophyll b (Chl b), and chlorophyll a + b (Chl a + b), were assessed by using the method of Weng et al. (2020). A leaf sample (0.10 g) was collected from fresh leaves of soybean, made into small size, and transferred into a 15 ml centrifuge tube, which was filled with 10 ml of solution by 95.5% acetone and absolute ethyl alcohol in the ratio of 1:1. Then, the tube was stored at a dark place to avoid light for 24 h or until the color of the leaf sample was changed into white. The absorbance concentrations of Chl a, Chl b, and Chl a + b were measured at 645, 652, and 663 nm, respectively, using a UV-visible spectrophotometer.

### Photosynthetic Gas Exchange

Photosynthetic gas exchange was determined with a portable infrared gas analyzers (IRGAs)-based photosynthetic system (Li-6400, Li-Cor, Lincoln, NE, United States) between 8.30 a.m. and 10.30 a.m. Nine leaves were needed for each sample with the following arrangement: molar flow of air per unit leaf area was 400.00 mmol m^–2^⋅s, water vapor pressure into leaf chamber was 3.7 mbar, photosynthetically active radiation (PAR) at leaf surface was up to 978 mmol m^–2^⋅s, leaf temperature was ranged from 29.9 to 37.5°C, ambient temperature was ranged from 30.1 to 37.8°C, ambient CO_2_ concentration was 399.9 mol mol^–1^, and RH was 59.78%. The parameters of photosynthetic gas exchange consisted of net photosynthesis (A), transpiration (E), stomatal conductance (Gs), and intercellular CO_2_ concentration (Ci). The ratio between A and E was known as water use efficiency (WUE).

### Plant Nutrients

Dry matter of plant samples was obtained after being dried in an oven at 65°C for 48 h. The Kjeldahl method was used for the determination of total nitrogen content in plant tissues. Phosphorus content was determined by the colorimetric method. Potassium content was determined by the flame photometer method. All the methods and procedures were followed as described by Jiatong (2008).

### Soil Properties

Soil samples were collected at the same stages as plant samples. They were air-dried and then sieved to pass through 2 mm. Soil pH (H_2_O) was measured in a 1:5 soil to water ratio with pH digital apparatus. The Kjeldahl method was used for the determination of soil total nitrogen content. Total phosphorus was determined using the melting method. Soil total potassium was determined using the flame photometer method (Lu, 2000). Total organic carbon (TOC) was determined using Shimadzu TOC-VCSH high-sensitivity combustion TOC analyzer (Shimadzu Corporation, Japan) with a dry combustion method (Nelson and Sommers, 1996). The 1 M NH_4_OAc saturation method at pH7 was used to measure CEC (Jiatong, 2008).

The soil samples for the soil microbial community were restored in the fridge to keep fresh before the test. The phospholipid fatty acids (PLFAs) method was used for the measurement of soil microbial diversity. PLFAs were extracted from fresh sieved soil at the maturity stage. The procedures and standard methods of PLFA were followed as described by Zelles and Bai (1993), Zelles (1999), and Buyer and Sasser (2012). The recognized PLFAs were appointed into a microbial group, such as eukaryotes, poly-unsaturated fatty acids (Ringelberg et al., 1997; Zelles, 1999), Gram-positive bacteria (e.g., branched saturated fatty acids), Gram-negative bacteria (e.g., monounsaturated fatty acids) (Zelles, 1999), and protozoa labeled as 20:3 and 20:4 (Ringelberg et al., 1997; Buyer and Sasser, 2012).

### Statistical Analysis

The factorial ANOVA technique was used to analyze the data. Only the significant ANOVA results were fixed with the least significant difference (LSD) test with a probability of 0.05, using the statistical computer package programmer MSTAT-C (Russell, 1986). Graphics of the data was made using Microsoft Excel.

## Results

### Growth, Yield, and Yield Components

In the case of applying biochar, the plant height, shoot dry weight, root dry weight, LAI, leaf dry weight, and yield of soybean were higher than those of no biochar treatments.

**FIGURE 1:**
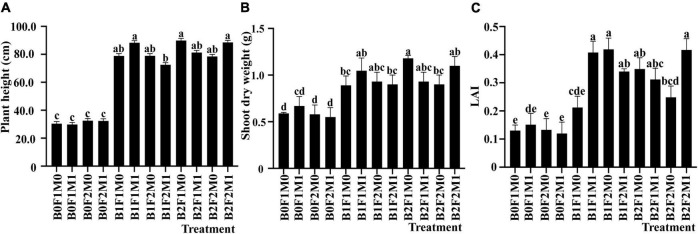
The effect of biochar, inorganic fertilizer, and organic fertilizer on plant height **(A)**, shoot dry weight **(B)**, and LAI **(C)** of soybean at vegetative stage. Bars marked by the same letter are not significantly different according to LSD-test (*P* ≤ 0.05).

During the vegetative stage, the application of biochar, inorganic, and organic fertilizers had significant effects on plant height and shoot dry weight of soybean. Compared with B0F1M0, B2F1M0 had the highest plant height, reaching 194.92% (Figure 1A). Compared with B0F1M0, the shoot dry weight of B2F1M0 treatment was increased by 100.21% (Figure 1B). All the growth indexes without biochar addition were the lowest.

During the vegetative stage, LAI was remarkably influenced by the application of biochar together with inorganic and organic fertilizers. Compared with B0F2M0, LAI under B1F2M0 increased by 212.43% (Figure 1C). LAI was only affected by biochar application at the beginning pod stage and full pod stage. Under B1 treatment, the maximum increase of LAI (806.71%) occurred at the beginning pod stage, and then gradually decreased until maturity.

**FIGURE 2:**
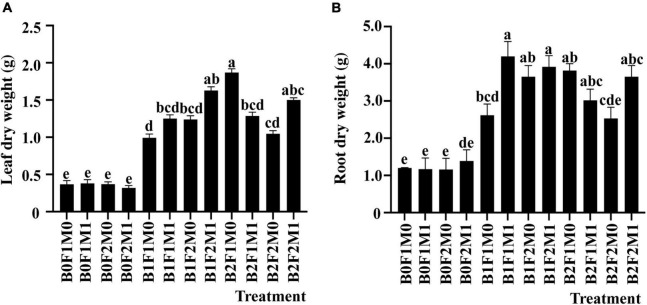
The effect of biochar, inorganic fertilizer, and organic fertilizer on leaf dry weight **(A)** and root dry weight **(B)** of soybean at the beginning pod stage. Bars marked by the same letter are not significantly different according to LSD-test (*P* ≤ 0.05).

At the beginning pod stage, the leaf dry weight and the root dry weight were significantly impacted by the application of biochar together with inorganic and organic fertilizers. The maximum leaf dry weight of B2F1M0 treatment was 409.81% higher than that of B0F1M0. However, there was no significant difference in leaf dry weight between B2F1M0 and B2F2M1, which was 343.32 and 300.55% higher than that of B0F1M0, respectively. At the same time, in all biochar treatments, the leaf dry weight of B1F1M0 was the lowest. However, B1F1M0 increased leaf dry weight by 170.84%, while B1F1M1 increased it by 239.37% compared with B0F1M0 (Figure 2A). The root dry weight of B1F1M1 was the highest, which was 246.28% higher than that of B0F1M0. The results showed that the root dry weight of B1F2M0, B1F2M1, B2F1M0, and B2F1M1 increased by 201.65, 223.97, 215.70, and 149.59%, respectively, compared with that of B0F1M0. Most of the root dry weight without biochar treatment was lower than that with biochar treatment (Figure 2B).

The yield and yield component were affected by biochar as the single treatment (Table 2). Compared with B0, the total pod number of B1 and B2 treatments increased by 85.26 and 108.14%, respectively, and the 100-seed weight increased by 91.89 and 78.90%, respectively. There was no significant difference between B1 and B2 in total pod number, pod dry weight, seed dry weight, and pod shell dry weight. Most notably, seed dry weight of B1 and B2 increased by 953.51 and 1072.23%, respectively, compared with B0.

**TABLE 2:** The effects of biochar, inorganic fertilizer, and organic fertilizer on yield and yield components of soybean.

Treatment	Total number of pod	Pod dry weight (g/plant)	Shell dry weight (g/plant)	100 seed weight (g)	Seed dry weight (g/plant)	HI
Biochar (B)	**	**	**	**	**	**
B0	5.08^b^	0.73^b^	0.47^b^	8.75^c^	0.27^b^	0.17^b^
B1	9.42^a^	4.41^a^	1.56^a^	16.79^a^	2.86^a^	0.44^a^
B2	10.58^a^	4.94^a^	1.77^a^	15.65^b^	3.18^a^	0.44^a^
Inorganic fertilizer (F)	ns	ns	ns	ns	ns	*
Organic fertilizer (M)	ns	ns	ns	ns	ns	*
B × F	ns	ns	ns	ns	ns	ns
B × M	ns	ns	ns	ns	ns	ns
F × M	ns	ns	ns	ns	ns	ns
B × F × M	ns	ns	ns	ns	ns	ns
CV(%)	38.26	37.8	36.17	8.97	34.99	16.07

*Mean values followed by the same letter within columns are not significantly different according to the LSD test (p ≤ 0.05).*

* and ** Significant difference at p = 0.05 and p = 0.01, respectively, and ns indicates no significant difference. CV is coefficient of variation (%).

### Chlorophyll Contents

In the case of applying biochar, the chlorophyll was higher than that of no biochar treatments.

During all the growth stages, there was generally no significant interaction for chlorophyll contents among biochar application, inorganic fertilizer, and organic fertilizer (Table 3). Biochar as a single treatment generally had an extremely significant effect on chlorophyll content. During the vegetative stage, B1 and B2 increased Chl b by 18.26 and 18.26%, and Chl a + b by 11.08 and 13.14%, respectively, compared with B0, but it did not significantly affect Chl a. During the beginning pod stage, under B1 and B2, Chl a increased by 3.6 and 2.37%, Chl b increased by 10.72 and 10.90%, and Chl a + b increased by 8.02 and 7.52%, respectively, compared with B0. During the full pod stage, under B1 and B2, Chl a increased by 11.57 and 13.42%, and Chl a + b increased by 17.51 and 15.71%, respectively, compared with B0.

**TABLE 3:** The effects of biochar, inorganic fertilizer, and organic fertilizer on chlorophyll content during plant growth.

Treatment	Vegetative stage (mg/g Fw)			Beginning pod stage (mg/g Fw)			Full pod stage (mg/g Fw)		
			
	Chl a	Chl b	Chl a+b	Chl a	Chl b	Chl a+b	Chl a	Chl b	Chl a+b
Biochar(B)	ns	**	**	**	**	**	**	ns	**
B0	2.53^a^	1.04^b^	3.88^b^	2.53^b^	1.10^b^	3.99^b^	2.16^b^	0.95^a^	3.37^b^
B1	2.63^a^	1.23^a^	4.31^a^	2.61^a^	1.24^a^	4.31^a^	2.41^a^	1.14^a^	3.96^a^
B2	2.62^a^	1.26^a^	4.39^a^	2.59^ab^	1.22^a^	4.29^a^	2.45^a^	1.10^a^	3.90^a^
Inorganic fertilizer (F)	ns	ns	ns	ns	ns	ns	ns	ns	*
Organic fertilizer (M)	ns	ns	ns	ns	ns	ns	ns	ns	ns
B × F	ns	ns	ns	ns	ns	ns	ns	ns	ns
B × M	ns	ns	ns	ns	ns	ns	ns	ns	ns
F × M	ns	ns	ns	ns	ns	ns	ns	ns	**
B × F × M	ns	ns	ns	ns	ns	ns	ns	ns	ns
CV(%)	3.51	12.86	9.26	2.75	10.59	6.51	8.33	19.53	13.49

*Mean values followed by the same letter within columns are not significantly different according to the LSD test (p ≤ 0.05).*

* and **Significant difference at p = 0.05 and p = 0.01, respectively, and ns indicates no significant difference. CV is coefficient of variation (%).

### Photosynthetic Gas Exchange

In the case of applying biochar, the intercellular CO_2_ concentration, transpiration, and stomatal conductance were higher than those of no biochar treatments.

During the full pod stage, the intercellular CO_2_ concentration, transpiration, and stomatal conductance were significantly impacted by the application of biochar together with inorganic and organic fertilizers (Table 4). The highest intercellular CO_2_ concentration was B0F1M1, which was 14.21% higher than that of B0F1M0. Compared with B0F1M0, the intercellular CO_2_ concentrations of B1F2M1, B1F1M0, B2F2M0, and B1F1M1 decreased by 9.45%, 4.52%, 0.73% and 0.49%, respectively.

**TABLE 4:** The effects of biochar, inorganic fertilizer, and organic fertilizer on photosynthetic gas exchange at the full pod stage.

Treatment	Intercellular CO_2_ concentration (μmol/mol)	Transpiration rate (mmol/m^2^⋅s)	Stomatal conductance (mol/m^2^⋅s)	Photosynthetic rate (μmol/m^2^⋅s)	Water use efficiency
B0F1M0	279.77^bc^	2.54^d^	0.13^e^	6.54^c^	2.56^bcde^
B0F1M1	319.55^a^	2.88^cd^	0.17^cde^	5.56^c^	1.95^e^
B0F2M0	296.33^ab^	2.86^cd^	0.15^cde^	5.99^c^	2.16^de^
B0F2M1	301.78^ab^	2.57^d^	0.13^de^	5.81^c^	2.25^cde^
B1F1M0	267.11^bc^	3.63^ab^	0.22^abc^	11.53^a^	3.20^abc^
B1F1M1	278.39^bc^	2.99^bcd^	0.17^bcde^	8.98^b^	3.01^abcd^
B1F2M0	297.11^ab^	3.42^abc^	0.21^abcd^	8.73^b^	2.54^bcde^
B1F2M1	253.33^c^	2.77^cd^	0.16^cde^	9.74^ab^	3.56^a^
B2F1M0	299.44^ab^	3.88^a^	0.25^ab^	9.50^b^	2.45^bcde^
B2F1M1	268.16^bc^	2.95^cd^	0.18^bcde^	9.67^ab^	3.27^ab^
B2F2M0	277.72^bc^	3.63^ab^	0.21^abcd^	10.38^ab^	2.90^abcde^
B2F2M1	284.55^abc^	3.73^a^	0.27^a^	11.47^a^	3.08^abcd^
Biochar (B)	*	*	**	**	**
Inorganic fertilizer (F)	ns	ns	ns	ns	ns
Organic fertilizer (M)	ns	*	ns	ns	ns
B × F	ns	ns	ns	ns	ns
B × M	ns	ns	ns	ns	ns
F × M	ns	ns	ns	ns	ns
B × F × M	*	*	*	ns	ns
CV(%)	7.99	12.56	23.02	20.22	21.03

*Mean values followed by the same letter within columns are not significantly different according to the LSD test (p ≤ 0.05).*

* and ** Significant difference at p = 0.05 and p = 0.01, respectively, and ns indicates no significant difference. CV is coefficient of variation (%).

The highest transpiration rate was observed under B2F1M0, which was 52.76% higher than that of B0F1M0. The increase in transpiration rate of B0F2M1 was the lowest, which was 4.72% higher than that of B0F1M0.

During the full pod stage, the change trend of stomatal conductance was consistent with that of transpiration rate. In general, the transpiration rate and stomatal conductance without biochar addition (B0F1M0, B0F1M1, B0F2M0, and B0F2M1) were lower than those with biochar addition. B2F2M1 increased stomatal conductance by 112.50% compared with B0F1M0. Compared with B0F1M0, B0F2M1 had the least increase in stomatal conductance (4.72%).

Low and high application of biochar could improve the photosynthetic rate and WUE. Compared with no biochar application, the photosynthetic rate of B1 and B2 increased by 66.41 and 71.70%, respectively, and the WUE increased by 38.18 and 31.22%, respectively.

### Plant Nutrients

Biochar as the only treatment had an effect on plant nitrogen content at the beginning pod, full pod, and maturity stages (Table 5). B2 and B1 significantly increased plant nitrogen content. The nitrogen content in stems of B0 was higher than that of B1 and B2 at the full pod stage.

**TABLE 5:** The nitrogen content (g kg^–1^) and protein contents (μg mg^–1^) in different parts of soybean at all growth stages as affected by biochar, inorganic, and organic fertilizer application.

Treatment	Vegetative	Beginning pod			Full pod				Maturity		
				
	Plant	Root	Stem	Leaf	Root	Stem	Leaf	Pod	Root	Stem	Pod
Biochar(B)	ns	**	ns	*	**	**	**	**	**	ns	**
B0	3.05^a^	3.30^b^	1.40^a^	3.30^b^	2.29^b^	2.33^a^	3.13^b^	1.98^b^	1.06^b^	1.98^a^	1.19^a^
B1	2.48^a^	3.78^a^	1.73^a^	3.78^a^	2.72^a^	1.66^b^	3.57^a^	4.77^a^	1.18^a^	1.74^a^	0.51^b^
B2	2.55^a^	3.80^a^	1.59^a^	3.80^a^	2.60^a^	1.71^b^	3.47^a^	4.76^a^	1.24^a^	1.70^a^	0.60^b^
Inorganic fertilizer (F)	ns	ns	ns	ns	ns	ns	ns	ns	ns	ns	ns
Organic fertilizer (M)	ns	ns	ns	ns	ns	ns	ns	ns	ns	ns	ns
B × F	ns	ns	ns	ns	ns	ns	ns	ns	ns	ns	ns
B × M	ns	ns	ns	ns	ns	ns	ns	ns	ns	ns	ns
F × M	ns	ns	ns	ns	ns	ns	ns	ns	**	ns	ns
B × F × M	ns	ns	ns	ns	*	ns	ns	ns	**	ns	ns
CV(%)	30.88	9.48	20.28	12.35	11.85	27.86	6.39	9.03	8.66	27.21	34.35

*Mean values followed by the same letter within columns are not significantly different according to the LSD test (p ≤ 0.05).*

* and ** Significant difference at p = 0.05 and p = 0.01, respectively, and ns indicates no significant difference. CV is coefficient of variation (%).

The plant phosphorus content was significantly impacted by the application of biochar together with inorganic and organic fertilizers only in shoots at the vegetative stage and in roots at the beginning pod stage (Table 6), while biochar had a greater effect on plant phosphorus content at other stages. Similar to the nitrogen content of soybean, the phosphorus content was significantly affected by biochar application throughout the growing period. The results showed that B2 and B1 were more able to induce P uptake by plants than B0. The phosphorus content in roots was the highest at the beginning pod stage and then decreased until the maturity stage. In addition, the phosphorus content in leaves was highest at the full pod stage. B0 showed the highest phosphorus content in the whole pod stage, while B2 and B0 exhibited more phosphorus content than B1 at the maturity stage.

**TABLE 6:** The phosphorus content in different parts of soybean at all growth stages as affected by biochar, inorganic, and organic fertilizer application (g kg^–1^).

Treatment	Vegetative		Beginning pod			Full pod				Maturity		
				
	Root	Stem	Root	Stem	Leaf	Root	Stem	Leaf	Pod	Root	Stem	Pod
Biochar(B)	ns	*	*	**	**	**	**	**	ns	**	**	ns
B0	0.63^a^	2.18^b^	2.36^ab^	1.24^b^	2.18^b^	1.05^b^	2.46^a^	1.93^b^	1.16^a^	0.36^b^	1.44^b^	0.92^a^
B1	0.65^a^	2.31^a^	2.44^a^	2.14^a^	2.31^ab^	1.14^b^	1.81^b^	2.75^a^	1.26^a^	0.69^a^	1.96^a^	0.88^a^
B2	0.54^a^	2.59^a^	2.18^b^	2.42^a^	2.59^a^	1.36^a^	1.97^b^	2.74^a^	1.43^a^	0.70^a^	2.13^a^	0.87^a^
Inorganic fertilizer (F)	ns	ns	ns	ns	ns	ns	ns	ns	ns	ns	ns	ns
Organic fertilizer (M)	ns	ns	ns	ns	ns	ns	ns	ns	ns	ns	ns	ns
B × F	ns	**	**	ns	ns	ns	ns	ns	ns	ns	ns	ns
B × M	ns	ns	**	ns	ns	ns	ns	ns	ns	ns	ns	ns
F × M	ns	*	*	ns	ns	ns	ns	ns	ns	ns	ns	ns
B × F × M	ns	**	**	ns	ns	ns	ns	ns	ns	ns	ns	ns
CV(%)	53	12.37	10.91	37.17	22.7	16.61	17.05	13.3	30.58	43.78	21.63	37.54

*Mean values followed by the same letter within columns are not significantly different according to the LSD test (P ≤ 0.05).*

* and ** Significant difference at p = 0.05 and p = 0.01, respectively, and ns indicates no significant difference. CV is coefficient of variation (%).

The plant potassium content was significantly impacted by biochar application after the vegetative stage. The application of biochar affected the potassium content in all parts of plants. The promoting effects of B2 and B1 on potassium absorption in different parts of plants were higher than those of B0 (Table 7).

**TABLE 7:** The potassium content in different parts of soybean at all growth stages as affected by biochar, inorganic, and organic fertilizer application (g kg^–1^).

Treatment	Vegetative		Beginning pod			Full pod				Maturity		
				
	Root	Stem	Root	Stem	Leaf	Root	Stem	Leaf	Pod	Root	Stem	Pod
Biochar (B)	**	*	**	**	**	**	**	**	**	*	**	*
B0	0.86^b^	0.81^b^	0.68^b^	0.83^b^	0.86^b^	0.63^b^	0.71^b^	0.64^b^	0.78^b^	0.62^b^	0.68^b^	0.79^b^
B1	1.02^a^	1.01^a^	0.86^a^	0.91^ab^	0.93^ab^	0.76^a^	0.85^a^	0.86^a^	0.90^a^	0.70^ab^	0.79^a^	0.85^a^
B2	1.02^a^	0.96^ab^	0.86^a^	0.99^a^	1.01^a^	0.76^a^	0.89^a^	0.90^a^	0.95^a^	0.74^a^	0.81^a^	0.94^a^
Inorganic fertilizer (F)	ns	ns	ns	ns	ns	ns	ns	ns	ns	ns	ns	ns
Organic fertilizer (M)	ns	ns	ns	ns	ns	ns	ns	ns	ns	ns	ns	ns
B × F	ns	ns	ns	ns	ns	ns	ns	ns	ns	ns	ns	ns
B × M	ns	ns	ns	ns	ns	ns	ns	ns	ns	ns	ns	ns
F × M	ns	ns	ns	ns	ns	ns	ns	ns	ns	ns	ns	ns
B × F × M	ns	ns	ns	ns	ns	ns	ns	ns	ns	ns	ns	ns
CV(%)	6	18.92	17.1	13.25	10.17	10.63	12.09	16.74	8.26	14.62	12.57	15.79

*Mean values followed by the same letter within columns are not significantly different according to the LSD test (p ≤ 0.05).*

* and ** Significant difference at p = 0.05 and p = 0.01, respectively, and ns indicates no significant difference. CV is coefficient of variation (%).

### Soil Fertility Properties

There was no significant interaction among biochar, inorganic fertilizer, and organic fertilizer application for soil pH (Table 8). However, the soil pH of biochar treatment (6.20–7.18) was higher than that of no biochar treatment (4.99–5.66).

**TABLE 8:** Soil TOC, CEC, and pH as affected biochar, inorganic, and organic fertilizer application.

Treatment	Total organic carbon(g/kg)	CEC[cmol(^+^)/kg]	pH
B0F1M0	6.12^d^	21.93^c^	5.66^cd^
B0F1M1	5.92^d^	23.23^bc^	5.55^cd^
B0F2M0	5.74^d^	24.89^bc^	5.58^cd^
B0F2M1	7.75^d^	24.63 bc	4.99^d^
B1F1M0	47.54^c^	33.52^abc^	6.29^abc^
B1F1M1	47.02^c^	34.18^abc^	6.39^abc^
B1F2M0	47.76^c^	36.54^ab^	6.20^bc^
B1F2M1	49.91^c^	35.27^ab^	6.33^abc^
B2F1M0	58.93^ab^	36.02^ab^	7.01^ab^
B2F1M1	61.69^a^	36.81^ab^	7.18^a^
B2F2M0	64.37^a^	40.41^a^	6.48^abc^
B2F2M1	51.83^bc^	39.20^a^	6.87^ab^
Biochar (B)	**	**	**
Inorganic fertilizer (F)	ns	ns	ns
Organic fertilizer (M)	ns	ns	ns
B × F	ns	ns	ns
B × M	ns	ns	ns
F × M	ns	ns	ns
B × F × M	*	ns	ns
CV (%)	12.41	6.62	5.11

*Mean values followed by the same letter within columns are not significantly different according to the LSD test (p ≤ 0.05).*

* and ** Significant difference at p = 0.05 and p = 0.01, respectively, and ns indicates no significant difference. CV is coefficient of variation (%).

Similar to soil pH, biochar, inorganic, and organic fertilizers had no effect on CEC. Only biochar as the single treatment affected CEC. Compared with B0, CEC increased by 61.02 and 47.36% under B2 and B1, respectively. The results showed that the application of biochar could increase soil CEC (Table 8).

The soil TOC of soybean was significantly affected by the application of biochar together with inorganic and organic fertilizers. The TOC of most biochar treatments was higher than those without biochar. The application of biochar, inorganic fertilizer, and organic fertilizer increased TOC by more than 600% compared with the application of inorganic fertilizer alone. TOC was the highest in B2F2M0, which was 951.80% higher than B0F1M0 and 908.64% higher than B0F1M0, but not statistically different with B2F1M1. The high rate of biochar induced more TOC than the low rate of biochar (Table 8).

**FIGURE 3:**
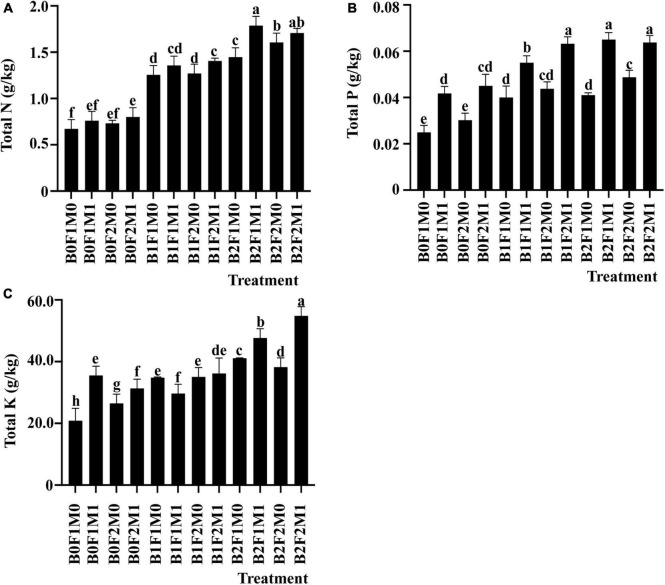
The effect of biochar application, inorganic fertilizer, and organic fertilizer on total N **(A),** total P **(B),** and total K **(C)** in soil at the maturity stage. Bars followed by the same letter are not significantly different according to LSD-test (*P* ≤ 0.05).

The soil total nitrogen, total phosphorus, and total potassium were significantly affected by the application of biochar together with inorganic and organic fertilizers. At the maturity stage, the soil total nitrogen content of B2F1M1 was the highest, which was 165.49% higher than that of B0F1M0. The soil total nitrogen content of B2F2M1 increased by 153.56% compared with B0F0M0. The total nitrogen of soil without biochar treatment was lower than that with biochar treatment (Figure 3A). B2F1M1 increased soil total phosphorus by 160.23%, and B1F1M1 increased soil total phosphorus by 120.45% compared with B0F1M0 (Figure 3B). The soil total potassium content of B2F2M1 was the highest, which was 162.62% higher than that of B0F1M0. The soil total potassium content of B2F1M1 was 128.41% higher than that of B0F1M0. The soil total potassium content of other treatments increased by less than 100% (Figure 3C).

### Soil Microbial Community

**FIGURE 4:**
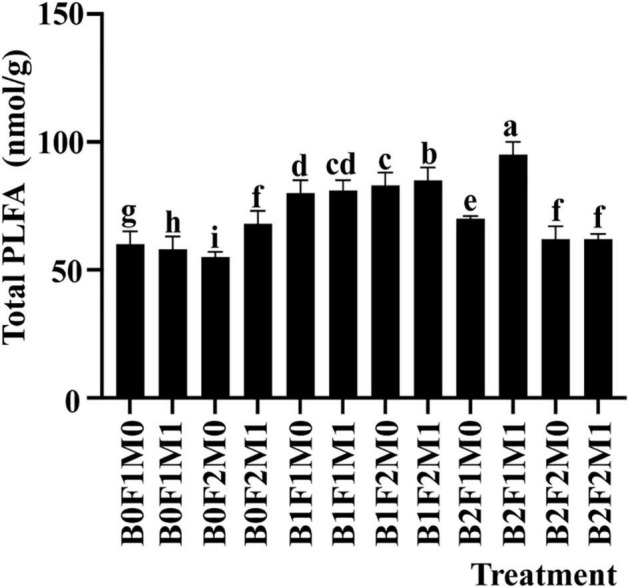
The effect of biochar application, inorganic fertilizer, and organic fertilizer on total PLFA in soil at beginning pods stage. Bars followed by the same letter are not significantly different according to LSD-test (*P* ≤ 0.05).

The microbial diversity in soil planted with soybean was significantly impacted by the application of biochar together with inorganic and organic fertilizers. Under B2F1M1, the total PLFA increased by 65.22% compared with B0F1M0 (Figure 4). The results showed that the total PLFA of half inorganic fertilizer treatment was higher than that of full inorganic fertilizer treatment, and the total PLFA of soil with organic fertilizer was higher than that without organic fertilizer.

**FIGURE 5:**
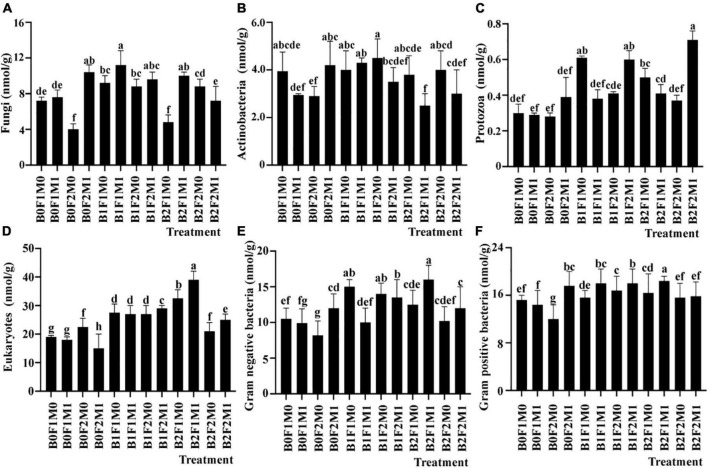
The effect of biochar application, inorganic fertilizer, and organic fertilizer on fungi **(A)**, actinobacteria **(B)**, protozoa **(C)**, eukaryotes **(D)**, gram-negative bacteria **(E),** and gram-positive bacteria **(F)** in soil at beginning pods stage. Bars followed by the same letter are not significantly different according to LSD-test (*P* ≤ 0.05).

Compared with B0F1M0, the development of fungi under B1F1M1 increased by 56.89%, and the development of fungi under B0F2M1, B1F2M1, and B2F1M1 increased by 46.32, 38.85, and 45.86%, respectively (Figure 5A). The results showed that a low biochar application rate had the greatest promoting effect on the soil fungal community.

The improvement of actinomycetes by biochar, inorganic, and organic fertilizer treatments was the same as that of fungi. Under B1F1M1 treatment, the development of actinomycetes increased by 22.22% compared with B0F1M0 (Figure 5B). The results showed that low biochar combined with inorganic and organic fertilizers could induce actinomycetes.

Compared with B0F1M0, the growth rate of protozoa under B2F2M1 was the highest, reaching 128.13%. Compared with B1F2M1 and B1F1M0, the growth rates of protozoan were 96.87 and 93.75%, respectively. The results showed that the rate of increase was lowest in the soil without biochar addition (Figure 5C).

The composition of eukaryotes was the same as that of total PLFA. The number of eukaryotes under B2F1M1 increased by 120.41%, while that under B2F2M1 only increased by 31.29% compared with B0F1M0. The results showed that a high rate of biochar and a full rate of inorganic fertilizer reduced the number of eukaryotes. Meanwhile, compared with no organic fertilizer application, organic fertilizer application promoted the composition of eukaryotes (Figure 5D).

Similarly, biochar application also improved the eukaryotes of Gram-negative bacteria and Gram-positive bacteria. Compared with B0F1M0, Gram-negative bacteria and Gram-positive bacteria under B2F1M1 increased by 64.49 and 44.79%, respectively (Figures 5E,F). The structural population of Gram-positive bacteria was higher than that of Gram-negative bacteria under all the treatments.

## Discussion

### Growth, Yield, and Yield Components

In this study, it was shown that B1F1M1 significantly increased plant height, shoot dry weight, and root dry weight. This result was consistent with previous reports in rice (Wang et al., 2012) and oats (Schulz and Glaser, 2012), in which biochar had a beneficial effect on plant growth (Wang et al., 2014). In addition, Schulz et al. (2013) explained that the combination of biochar and organic fertilizer could activate plant growth and increase fertilizer use efficiency. This study showed that biochar, as an adsorbent for organic and inorganic fertilizers, increased crop yield and reduced the requirement of fertilizers (Widowati and Asnah, 2014).

This study also showed that single biochar application increased yield and yield components compared with no biochar application, in which seed dry weight increased by 10 times, and 100-grain weight increased by 91.89%, being consistent with previous studies in wheat (Vaccari et al., 2011), maize (Chen et al., 2021), soybean (Haider et al., 2021), and rice (Nan et al., 2019). The meta-analysis of Verheijen et al. (2009) predicted that the addition of biochar could increase crop yield by 12% in short term.

Crop productivity depends on its photosynthetic capacity and photosynthetic area (Jones, 2014). LAI was affected by low rate biochar, inorganic fertilizer, and organic fertilizer. At the beginning pod stage, LAI was only affected by biochar application. LAI under low rate application of biochar reached the maximum at the beginning pod stage and then decreased gradually until maturity stage. This result was consistent with the description of Hunt et al. (2002).

### Chlorophyll Contents

During the whole growth stage, biochar affected the chlorophyll content in soybean plants. The results showed that the chlorophyll content of soybean with biochar was higher than that without biochar, whether low or high rate. Dudeja and Chaudhary (2005) explained that the change of chlorophyll content was the result of soil nutrient changes. The presence of inorganic and organic fertilizers improved soil nutrients, thus affecting the chlorophyll content. Chlorophyll is crucial for photosynthesis, which enables plants to absorb energy from light (Hikosaka, 2021). It was found that the chlorophyll content in the treatments without biochar was higher than that in the treatments with biochar. This proved that the existence of biochar could improve the available nutrients in the soil and thus increase chlorophyll content in plants.

### Photosynthetic Gas Exchange

This study showed that the photosynthetic gas exchange properties of soybean plants were improved by biochar treatments. The high rate of biochar application together with half rate of inorganic and organic fertilizers was conducive to the growth environment of plants at the full pod stage. Mafakheri et al. (2010) explained that plants growing in unsuitable environments exhibited a decrease in stomatal conductance. Subsequently, CO_2_ fixation decreased, and photosynthetic rate decreased, resulting in the decrease of assimilating yield required for plant growth and yield. This study also showed that biochar combined with inorganic and organic fertilizers increased stomatal conductance and intercellular CO_2_ concentration, thereby increasing photosynthetic rate and WUE, and ultimately promoting plant growth and increasing yield.

### Plant Nutrients

This study found that biochar application increased the uptake of nitrogen, phosphorus, and potassium in leaves and roots of soybean. Biochar promoted nutrient uptake by increasing nutrient utilization and reducing fertilizer leaching (Yamato et al., 2006). This result is similar to previous studies conducted by Widowati and Asnah (2014) who found that biochar application increased potassium uptake by 128%. Xiang et al. (2017) explained that biochar application improved soil environment, and stimulated root length, thus being conducive to the absorption of soil water and nutrients.

### Soil Fertility Properties

The TOC content is one of the important soil properties related to soil quality (FAO, 2017). This study showed that the application of biochar could promote the TOC content of soil. This is because biochar is a solid material containing carbon (Joseph et al., 2010; Wilson, 2014). The combination of biochar with inorganic and organic fertilizers had a stronger inducing effect on TOC, which could be explained by the ability of biochar as a soil amendment to improve soil quality (Oram et al., 2014). Yan et al. (2019) showed that biochar has an impact on the purple soil carbon environment and soil microbial abundance, which in turn may improve soil carbon pools. This study showed that under a high rate of biochar application, TOC content of soil was higher than that under a low rate of biochar application, being similar to the previous study conducted by Schulz et al. (2013), in which biochar was applied at a rate of 90 Mg ha^–1^ to induce plant growth and increase carbon storage potential. This proved that biochar, inorganic, and organic application could significantly increase soil organic carbon content.

One of the parameters for soil fertility is CEC (Wilson, 2014; Nanda et al., 2016). This study showed that biochar as a single treatment had an impact on CEC. Higher CEC indicates a higher ability to maintain and transfer nutrients, which is crucial for plant development (Arif et al., 2017). Li et al. (2021) noticed that a high dosage of biochar increased the surface area and CEC of soil. Osman (2013) explained that CEC value depended on soil pH, and it was shown that biochar was needed as a soil conditioner for low pH and low CEC soil; therefore, the soil pH and nutrient availability were improved by increasing CEC.

This study showed that biochar combined with inorganic and organic fertilizers could improve soil nutrient characteristics. It was worth noting that soil total nitrogen of biochar treatment was higher than that of no biochar treatment, whether without organic fertilizer or with half or total amount of organic fertilizer. Tian et al. (2020) showed that the use of biochar can reduce fertilizer application in purple soil regions. Yamato et al. (2006) reported that the application of bark biochar combined with chemical fertilizer improved soil total nitrogen, available phosphorus, and pH compared with the application of nitrogen only. This study also showed that the presence of biochar, inorganic fertilizer, and organic fertilizer had a certain promoting effect in increasing soil total potassium content (mainly B2F2M1 and B2F1M1 treatments) and total phosphorus (B1F1M1 treatment). Cui et al. (2011) explained that biochar application could accelerate the availability of phosphorus in the soil, as biochar treatment could reduce phosphorus adsorption on iron oxides and provide nutrients for plants. The results showed that the soil phosphorus content under treatments without organic fertilizer was lower than that under treatments with organic fertilizer. Carter et al. (2013) also showed that the combined application of biochar and organic fertilizer changed soil structure and improved soil chemical properties. Tian et al. (2021) showed that biochar improved soil quality in purple soils, thereby promoting increased rapeseed yield. This study also proved that the application of biochar combined with organic and inorganic fertilizers greatly improved soil quality.

### Soil Microbial Community

The porous structure of biochar can improve the ability to adsorb soluble organic matter and gases, promote microbial habitats, and protect microorganisms from predators (Thies and Rillig, 2009). This study showed that biochar had a significant effect on soil microbial communities. The application of biochar affected the soil organic matter, soil carbon, CEC, and soil pH, thereby affecting the soil microbial community structure. The total PLFA, fungi, Gram-negative bacteria, Gram-positive bacteria, and protozoa in soil microbial communities under B2F1M1 were the highest. Soil microbial communities were affected by high concentrations of biochar and half inorganic fertilizer, but organic fertilizer was needed to promote soil microbial communities. Marschner et al. (2003) pointed out that in agricultural practices, the use of organic and inorganic fertilizers to increase the nutrient availability for plants could also stimulate soil microorganisms. At the same time, the surface area and pore of biochar could be used as a habitat for microbial colonization (Lehmann et al., 2011; Steiner et al., 2016).

In this study, the purple soil nutrient content and pH value increased under the application of biochar together with inorganic and organic fertilizers. Steiner et al. (2016) pointed out that soil pH was one of the key parameters to induce microbial community formation. After biochar application, the increase of soil pH as a liming effect could significantly promote the biological activity of beneficial microorganisms (Ameloot et al., 2013; Fageria, 2013).

## Conclusion

In this study, the application of biochar together with inorganic and organic fertilizers escalated the plant growth of soybean. There was no significant difference between high rate and low rate of biochar on plant growth, while the combination of biochar with half rate of inorganic and organic fertilizers improved plant growth more significantly than the treatments of full rate of inorganic fertilizer without organic fertilizer or biochar. Biochar significantly increased yield and yield components.

The presence of biochar, whether high or low rate, together with half inorganic and organic fertilizers, increased photosynthetic gas exchange and nutrient uptake of soybean, and nutrients contents and microbial community in soil. There was no significant difference for all observed plant and soil parameters between low rate and high rate of biochar, while the combination of biochar with half rate of inorganic and organic fertilizers significantly improved plant and soil parameters. The existence of biochar could reduce the application rate of inorganic fertilizer when combined with organic fertilizer.

## Data Availability Statement

The original contributions presented in the study are included in the article/supplementary material, further inquiries can be directed to the corresponding author/s.

## Author Contributions

ML: writing – original draft. CL: data curation. SM, QM, JG, and FW: investigations. LW: writing – review and editing. All authors: contributed to the article and approved the submitted version.

## Conflict of Interest

The authors declare that the research was conducted in the absence of any commercial or financial relationships that could be construed as a potential conflict of interest.

## Publisher’s Note

All claims expressed in this article are solely those of the authors and do not necessarily represent those of their affiliated organizations, or those of the publisher, the editors and the reviewers. Any product that may be evaluated in this article, or claim that may be made by its manufacturer, is not guaranteed or endorsed by the publisher.
